# Histomorphometry of Ossification in Functionalised Ceramics with Tripeptide Arg-Gly-Asp (RGD): An In Vivo Study

**DOI:** 10.3390/life12050761

**Published:** 2022-05-20

**Authors:** Filippo Migliorini, Hanno Schenker, Nicola Maffulli, Frank Hildebrand, Jörg Eschweiler

**Affiliations:** 1 Department of Orthopaedic, Trauma and Reconstructive Surgery, RWTH University Hospital, 52074 Aachen, Germany; migliorini.md@gmail.com (F.M.); hschenker@ukaachen.de (H.S.); fhildebrand@ukaachen.de (F.H.); joeschweiler@ukaachen.de (J.E.); 2 Department of Medicine, Surgery and Dentistry, University of Salerno, 84081 Baronissi, Italy; 3 School of Pharmacy and Bioengineering, Keele University Faculty of Medicine, Stoke on Trent ST4 7QB, UK; 4 Barts and The London School of Medicine and Dentistry, Centre for Sports and Exercise Medicine, Mile End Hospital, Queen Mary University of London, London E1 4DG, UK

**Keywords:** functionalised ceramic, RGD, implantology, ossification

## Abstract

The present study investigated the osseointegration promoted by functionalised ceramics with peptide Arg-Gly-Asp (RGD) in a rabbit model in vivo. Histomorphometry of the RGD functionalised ceramic implants was conducted by a trained pathologist to quantify the amount of mature and immature ossification at the bone interface, and then compared to titanium alloy implants. The region of interest was the area surrounding the implant. The percentage of ROI covered by osteoid implant contact and mature bone implant contact were assessed. The presence of bone resorption, necrosis, and/or inflammation in the areas around the implant were quantitatively investigated. All 36 rabbits survived the experimental period of 6 and 12 weeks. All implants remained in situ. No necrosis, bone resorption, or inflammation were identified. At 12 weeks follow-up, the overall mean bone implant contact (*p* = 0.003) and immature osteoid contact (*p* = 0.03) were improved compared to the mean values evidenced at 6 weeks. At 6 weeks follow-up, the overall osteoid implant contact was greater in the RGD enhanced group compared to the titanium implant (*p* = 0.01). The other endpoints of interest were similar between the two implants at all follow-up points (*p* ≥ 0.05). Functionalised ceramics with peptide RGD promoted ossification in vivo. The overall osteoid and bone implant contact improved significantly from 6 to 12 weeks. Finally, RGD enhanced ceramic promoted faster osteoid implant contact in vivo than titanium implants. Overall, the amount of ossification at 12 weeks is comparable with the titanium implants. No necrosis, bone resorption, or inflammation were observed in any sample.

## 1. Introduction

Metallic implants are commonly used in musculoskeletal medicine. Given its biocompatibility and ossification potential, alongside relatively lower elasticity module and breaking strength, titanium is the most commonly used component of prosthetic implants [1,2,3,4,5]. However, titanium alloys may be responsible for hypersensitivity reactions, which may compromise implant longevity. When low-grade infection and other mechanical problems have been excluded, symptoms, such as pruritus, pain, effusion, erythema, and hypersensitivity reactions, should be taken into consideration [6]. Ions released by corrosion of metallic wear debris may impair ossification and metal particles can be found in the soft tissues surrounding the implant [7]. Particles and ions may become clinically relevant for sensitive patients. According to the 2016 Australian Arthroplasty Register, approximately 2% of revision TKAs are consequent to metal-related pathology [8]. In selected patients with hypersensitivity, non-metallic implants may be used with unpredictable results [9]. Current research to develop alternatives to metal alloys is ongoing. In this context, ceramic implants offer several advantages: hardness and wear resistance; light weight; low modulus of elasticity; outstanding resistance to creep and compressive stress; and lack of artefacts at imaging [10,11,12]. Although it reduces particle release, the risk of rejection, and implant loosening, the biological inactivity of ceramic impairs the integration of the implant into the surrounding bone tissue [13,14,15]. 

We developed a coating methodology using high-performance ceramic biologically activated with a silicate coating on alumina (AL_2_O_3_) and coated with peptide Arg-Gly-Asp (RGD) [16,17,18,19]. This study investigated the osseointegration provided by ceramic functionalised with the RGD peptide in vivo. The peptide RGD, derived from fibronectin in the extracellular matrix, increases cell attachment with optimal stable bioactivity and has been postulated to enhance implant osteointegration [20,21,22]. To the best of our knowledge, no previous study has evaluated the osteointegration of activated ceramic implants enhanced with the RGD peptide. We, therefore, conducted histomorphometry studies around the distal, proximal, medial, and lateral sides of the RGD functionalised ceramic implants to quantify the amount of mature and immature ossification within the bone interface. We hypothesised that ceramic implants functionalised with the RGD peptide promote ossification and transplant integration. The osteointegration of RGD functionalised ceramics was compared to that provided by commercially available titanium alloy implants.

## 2. Material and Methods

### 2.1. Sample Preparation

Sandblasted titanium implants by Fa. Zimmer Biomet Deutschland GmbH (Neu-Ulm, Germany), with a diameter of 5.5 mm and length of 8 mm, were used for surgery. Activated ceramics were manufactured at the Department of Dental Materials Science and Biomaterial Research of the RWTH University Aachen, Germany. The process to obtain active ceramic samples has been described in detail [23]. Briefly, standard Al_2_O_3_ ceramic based cylinders were used. Plasma-enhanced chemical vapor deposition (PE-CVD) was performed to facilitate the coupling of stable organosilane monolayers on the monolithic Al_2_O_3_ ceramic based cylinders [24]. These cylinders were activated using silicon suboxide (SiOx), which was deposited on the polished and Al_2_O_3_ ceramic based cylinders [25]. Activated ceramic cylinders were then air-dried, cured at 80° for 45 min, and stored in liquid nitrogen until use. The day before surgery, the activated ceramics were coated with RGD. RGD was purified using size exclusion centrifugation and coated over the activated ceramic cylinders using crosslinkers bis(succinimidyl) suberate (BS3, Thermo Fisher, Woodward Austin, TX, USA) as received [26]. Purity was confirmed via antibody detection (monoclonal clone antibody anti-RGD, R&D Systems, Germany) and osteogenic induction capabilities via Human Mesenchymal Stem Cells (hMSC) [27]. Protein concentration was measured using a bicinchoninic acid assay (BCA, Thermo Fisher, Woodward Austin, TX, USA) [28]. All experiments were performed in triplicate. More detailed information on the RGD coating process can be found in a previous report [29].

### 2.2. Surgical Procedure

**Figure 1:**
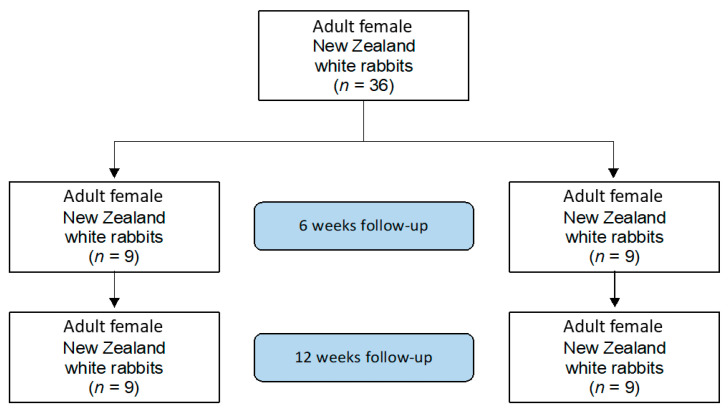
Flow chart of the study set-up.

This study was conducted in 2016 according to the Animal Welfare Act of the Federal Republic of Germany. This study was approved by the Federal Office for Nature, Environment and Consumer Protection (Landesamt für Natur, Umwelt und Verbraucherschutz, LANU) of North Rhine-Westphalia, Federal Republic of Germany (Approval ID: 84-02.04.2016.A434). For the study, 36 adult female New Zealand white rabbits with a minimum weight of three kilograms were used. Rabbits were randomly allocated into four groups (Figure 1). 

**Figure 2:**
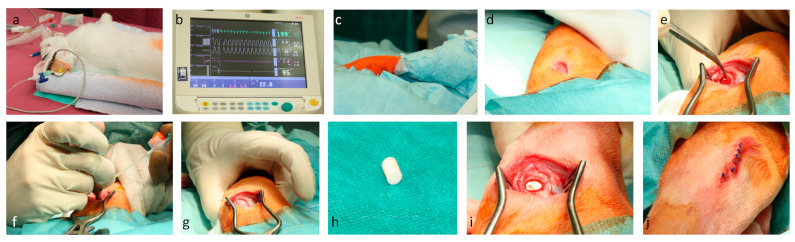
General anaesthesia (**a**), under monitoring (**b**), sterile dressed surgical site (**c**), lateral femoral skin incision (**d**), preparation of lateral femoral aspect (**e**), water cooled drilling (**f**), extraction of the bony cylinder (**g**), ceramic cylinder (**h**), sample in situ (**i**), sutured wound (**j**).

Before the surgical procedure, general anaesthesiassss was provided with 0,1 mL/mg/kg bodyweight Medetomidin (Domitor) combined with 0.2 mL Ketamin (Narketan) 10% via subcutaneous injection (Figure 2a,b). The surgical site was shaved, disinfected with iodine and ethanol, and draped in a sterile fashion (Figure 2c). Before incision, 10 mg/kg bodyweight Enrofloxacin was injected subcutaneously. A skin incision was performed over the right lateral femoral condyle (Figure 2d). After preparation through fascia and muscle, the lateral femoral condyle was exposed (Figure 2e). The lateral collateral ligament (LCL) was identified and spared. A unicortical drillhole with a 5.5 mm trephine was prepared under continuous irrigation to avoid thermal necrosis (Figure 2f). After extraction of the bony cylinder (Figure 2g), either a titanium or silanised ceramic cylinder (Figure 2h) was inserted in a press fit fashion (Figure 2i). Attention was given to avoid the lesion of the knee capsule. After irrigation with saline solution, the tissues were closed in layers. Finally, the skin was stapled and sealed with chelated silver spray (Figure 2j). For the first three days after surgery, 4 mg/kg bodyweight Carprofen was applied every 24 h. At 6 and 12 weeks postoperatively, the animals were euthanised with 2 mL/kg bodyweight Natriumpentobarbital (160 mg Natriumpentobarbital/mL), and the femoral condyles were harvested. 

### 2.3. Sample Preparation

The femoral condyles were harvested en bloc. Fixation was performed over 12 days with 4% paraformaldehyde followed by an alcohol series with ethanol of 50–100% and xylol. The specimens were embedded in Technovit^®^ 9100 Fa. Heraeus Kulzer. Finally, coplanar thin cuts (60–70 µm) of the specimens were made with a diamond band saw Exakt 300CL. Grinding of titanium implants was performed with sandpaper, whereas silanised ceramics were ground with diamond paper. All specimens were stained with haematoxylin eosin, trichrome, and toluidine. Histomorphometry was conducted by a trained pathologist with an OLYMPUS digital microscope DSX-1000 and stream desktop software (Olympus, Hamburg, Germany). 

### 2.4. Histomorphometry 

**Figure 3:**
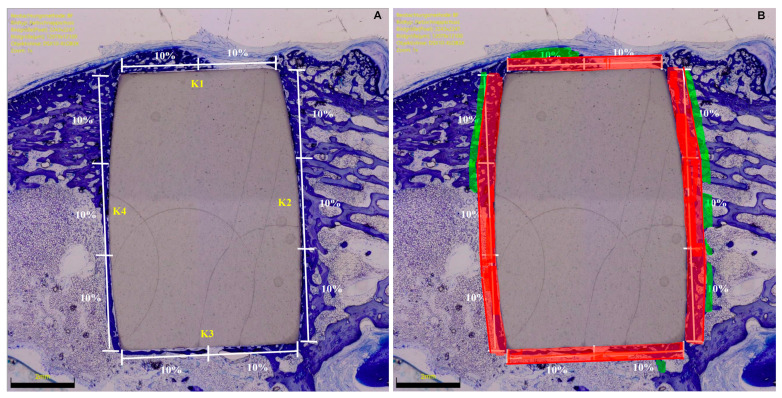
(**A**) Microscopic evaluation strategy of the BIC: K2 and K4 (longer sides) accounted for 60% (30% each) and the K1 and K3 (shorter sides) for 40% (20% each). (**B**) Region of interest.

At microscopic evaluation, the implant sides were divided into four subsections: lateral (K1), distal (K2), medial (K3), and proximal (K4). The region of interest (ROI) represented the percentage of the surrounding area of the implant (red zones) which was analysed (Figure 3). 

**Figure 4:**
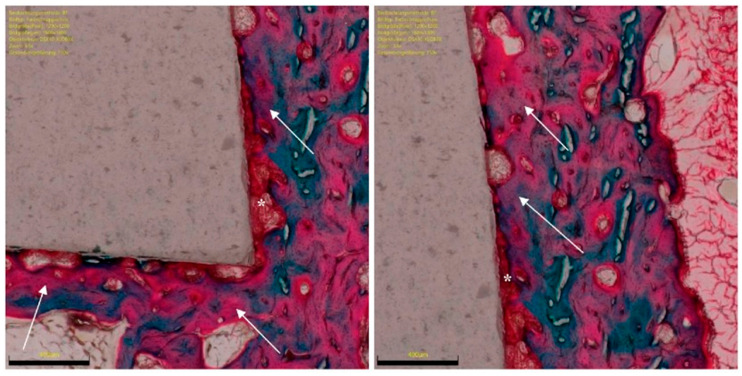
RGD functionalised ceramic implant with surrounding new bone formation (green to pink, arrows), resident bone (green) and osteoid (red, *), adjacent soft tissue. Section preparations in trichrome staining, each magnified 150×.

Bone density was measured within each ROI by evaluating the percentage of area filled with mineralized bone. The percentage of ROI covered by immature and unmineralized bone matrix, the osteoid implant contact (OIC), was also quantified (Figure 4). Mature bone implant contact (BIC) was assessed by analysing the length of mineralized bone with direct implant contact as a percentage (Figure 3). The presence of bone resorption, necrosis, and/or inflammation were quantitatively identified and classified: 0 (none), 1 (minimal), 2 (low), 3 (moderate), and 4 (severe).

### 2.5. Outcomes of Interests

We aimed to investigate the potential of osseointegration of ceramic RGD enhanced implants in comparison to standard titanium implants. Hence, OIC, BIC, and the presence of bone necrosis, bone resorption, and/or inflammation in the ROI were quantitatively assessed.

### 2.6. Statistical Analysis

The IBM SPSS (version 25) was used for the statistical analyses. For descriptive statistics, mean and standard deviation were calculated. For continuous data comparison, the mean difference effect measure was adopted, with standard error (SE) and T-value. The confidence interval (CI) was set at 95% in all the comparisons. The *t*-test was performed, with values of *p* < 0.05 considered statistically significant.

## 3. Results

All 36 rabbits survived the experimental period of 6 and 12 weeks. No wound dehiscence was reported. At euthanasia, no clinical signs of inflammation or adverse tissue reactions were observed. All implants remained in situ. At baseline, rabbits had a mean weight of 3456.2 ± 243.4 mg. At the last follow-up, rabbits had a mean weight of 4001.8 ± 345.6 mg (+545.6 mg; *p* < 0.0001). 

### 3.1. Ossification from 6- to 12-Weeks Follow-up of the RGD Enhanced Implants

No necrosis, bone resorption or inflammation were found in any sample. At 12 weeks follow-up, the overall mean BIC (*p* = 0.003) and IOC (*p* = 0.03) were improved compared to the mean values at 6 weeks (Table 1).

### 3.2. Comparison of RGD Enhanced Versus Titanium Implants

At 6 weeks follow-up, the overall OIC was greater in the RGD enhanced group compared to the titanium implant (*p* = 0.01) (Appendix A Table A1). The other endpoints of interest were similar between the two implants at all follow-ups (*p* ≥ 0.05). The results of the quantitative analyses are shown in detail in Table 2.

## 4. Discussion

**Figure 5:**
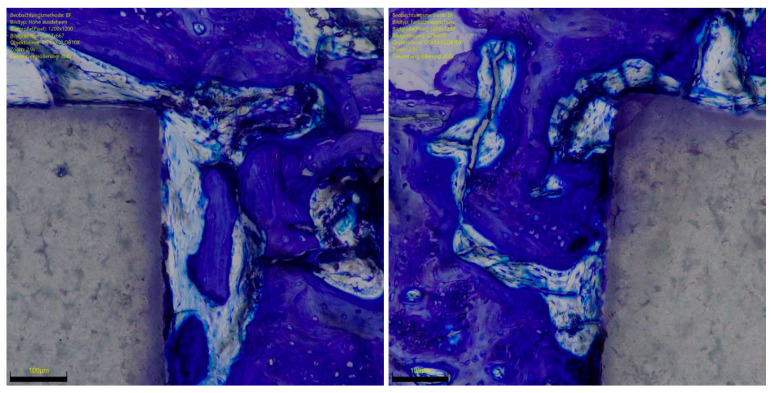
ROI of RGD functionalised ceramic implant with toluidine blue. Contour irregularities and porosities colonised by osteoblasts and osteocytes.

**Figure 6:**
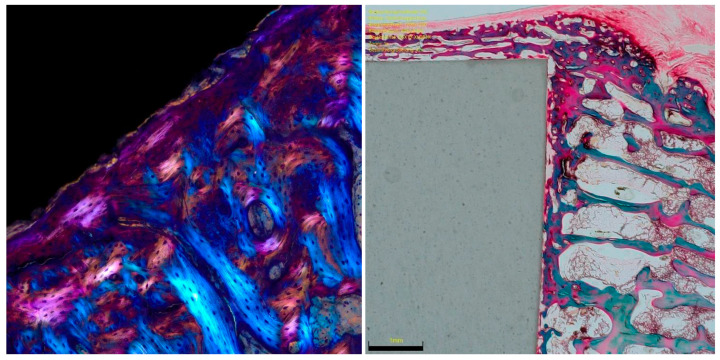
**Left**: Titanium implant in polarised light toluidine blue staining with adjacent newly formed bone, polarised optically magenta-orange and local bone blue. **Right**: RGD functionalised ceramic implant in trichrome staining with adjacent new bone formation in red-green and local bone in green.

Ceramic implants have several advantages, such as their high hardness and wear resistance, light weight, low modulus of elasticity, outstanding resistance to creep and compressive stress, and no artefacts at imaging [10,11,12]. Functionalised ceramics with RGD biologically activate the implant surface, enhancing the interaction at the bone implant interface, thus preserving the overall structure and characteristics of the ceramic. The present study confirmed our hypothesis that functionalised ceramic implants enhanced with peptide RGD promote in vivo ossification (Figure 5 and Figure 6). The overall osteoid and bone implant contact improved significantly from 6 to 12 weeks. Finally, RGD enhanced ceramics promoted faster osteoid implant contact in vivo than titanium implants. Overall, the amount of ossification at 12 weeks is comparable between the two implants. No necrosis, bone resorption, or inflammation was observed in any sample at any follow-up. 

The overall BIC improved from 6 to 12 weeks. This improvement was especially evident around the medial and distal portions of the bone implant interface. We hypothesised that the proximity to the cortical bone of the medial epicondyle may stimulate greater new bone formation. This assumption is also supported by the greater osteoid bone formation in this area. The vascular supply of the distal femur in rabbits arises from the epiphyseal vessels, and the epicondyle is supplied by arteries from the intercondylar notch and epicondyle regions [30,31]. This favours the blood supply into the distal part of the epicondyle, and, given its terminal anatomy, impairs the vascularisation of the distal epiphysis. 

Bone is dynamic and undergoes several changes and adaptations. In end stage degenerative or traumatic ailments, implants are commonly used to restore function. Such implants can be permanent or temporary. In case of permanent implants (e.g., arthroplasty), bone integration of the implant to the surrounding bone is crucial to ensure their longevity. The interaction between bones and surrounding tissue is continually regulated by a variety of complex mechanisms. Bone marrow ensures the maintenance of the modelling processes within the bone by regulating cellular migration [32]. Depending on the physical and chemical properties, a foreign body (such as an implant in bone tissue) may produce an immune response [33,34,35]. The coagulation cascade, the activation of the complement system, and the activation of platelets and immune cells are critical in regulating immune response [36,37,38,39,40,41,42,43,44]. Coagulation and complement interact synergistically and modulate the initial contact with the surface of the biomaterial [45]. In addition to the immediate immunological reaction, the interaction of implants and bone tissue undergo sequential phases: (1) adsorption of serum and proteins to the implant surface immediately after implantation, activation, proliferation, and adhesion of mesenchymal stem cells and the production of extracellular matrix; (2) osteogenic differentiation of the adhesive mesenchymal stem cells; and (3) calcification of the matrix and the remodelling of the newly formed bone based on the applied loads [35]. The mesenchymal stem cells adhere mainly to the RGD sequences located within the adsorbed proteins, which serve as ligands for the integrin contained within the phospholipidic membrane [46]. The amino acid sequence consisting of arginine (R), glycine (G), and aspartic acid (D) bind the α and β subunits of the trans-membrane heterodimeric integrin [47,48,49,50]. The intracellular signalling pathway activated by the RGD integrin binding is extremely varied [51]. Among them, the non-receptor tyrosine kinase FAK (Focal Adhesion Kinase, also PTK2) is activated [52,53], and influences the adhesion of the cell on the extracellular matrix and cell motility in a phosphorylated status. These features may explain the faster osteoid implant integration observed in the first six weeks of the RGD functionalised ceramics.

Several coating processes to functionalise the surface of ceramic have been developed with promising pre-clinical and clinical results [54,55,56,57]. Previous studies conducted in rabbit models used heterogeneous coating methodologies, follow-up, analyses, and implantation sites (Table 3). The current evidence on the clinical applications of functionalised ceramic is still limited, and translational studies are required to clarify the best coating process. 

Our results confirmed that the in vivo osteointegration was similar to that of the titanium implants. However, these results must be considered in light of some limitations. Firstly, between-species differences in histology, cytology, and functional anatomy may impact the reliability of the clinical applicability of our conclusions. The translational potential of each animal model to human clinical conditions is different; however, being reproducible, low cost, and easy to handle, rabbits are one of the most used animal models for in vivo experimentations. Biomechanical evaluation of the bone formed—which could provide a comprehensive understanding of bone repair—was not conducted. In the present study, the specimens were stained with haematoxylin eosin, trichrome, and toluidine. Further investigations are needed to evaluate dynamic new bone formation using other methodologies, for example fluorescent with calcein green. Only female rabbits were included in the present investigation. This may introduce biases and between-gender differences were not considered. These results encourage further translational studies to validate our findings in a clinical setting.

## 5. Conclusions

Functionalised ceramic enhanced with RGD peptide promoted in vivo osteointegration. The overall osteoid and bone implant contact improved significantly from 6 to 12 weeks. Finally, RGD enhanced ceramics promoted faster osteoid implant contact in vivo than titanium implants. Overall, the amount of osteointegration at 12 weeks is comparable with that of titanium implants. No necrosis, bone resorption or inflammation was observed in any sample at any follow-up point.

## Figures and Tables

**Table 1:** Ossification potential of RGD enhanced implants from 6- to 12-weeks (BIC: bone implant contact; OIC: osteoid implant contact; MD: mean difference; CI: confidence interval; SE: standard error).

Endpoint		6 Weeks	12 Weeks	MD	95% CI	SE	*t* Value	*p*
Lateral	BIC (%)	3.1 ± 3.4	3.9 ± 1.3	0.8	−0.41 to 2.01	0.61	1.32	0.2
	OIC (%)	0.9 ± 1.2	0.5 ± 0.9	−0.4	−0.90 to 0.10	0.25	−1.60	0.1
Distal	BIC (%)	12.1 ± 4.6	14.3 ± 5.8	2.2	−0.26 to 4.66	1.23	1.78	0.08
	OIC (%)	3.4 ± 3.0	6.0 ± 4.7	2.6	0.75 to 4.45	0.93	2.80	0.007
Medial	BIC (%)	6.8 ± 4.2	8.6 ± 3.2	1.8	0.05 to 3.56	0.88	2.05	0.04
	OIC (%)	3.2 ± 3.2	4.6 ± 4.3	1.4	−0.38 to 3.18	0.89	1.57	0.1
Proximal	BIC (%)	12.6 ± 3.5	13.1 ± 3.5	0.5	−1.14 to 2.14	0.83	0.61	0.5
	OIC (%)	5.0 ± 0.0	5.6 ± 1.8	0.6	0.00 to 1.20	0.30	2.00	0.05
Overall	BIC (%)	34.6 ± 7.9	39.9 ± 6.9	5.3	1.81 to 8.78	1.75	3.03	0.003
	OIC (%)	12.6 ± 6.0	16.8 ± 9.3	4.2	0.52 to 7.87	1.85	2.28	0.03

**Table 2:** Comparison of RGD enhanced versus titanium at 6- and 12-weeks follow-up (MD: mean difference). Negative mean difference indicates greater ossification in favour of the titanium group.

Endpoint		6 Weeks		12 Weeks	
		MD	*p*	MD	*p*
Lateral	BIC (%)	0.0	0.5	−1.0	0.05
	OIC (%)	0.9	0.02	1.1	0.4
Distal	BIC (%)	0.6	0.4	0.2	0.4
	OIC (%)	1.9	0.1	0.6	0.3
Medial	BIC (%)	1.5	0.2	1.1	0.05
	OIC (%)	3.5	0.004	1.3	0.05
Proximal	BIC (%)	1.7	0.3	1.0	0.4
	OIC (%)	0.0	1.0	0.7	0.09
Overall	BIC (%)	0.0	0.3	0.0	0.4
	OIC (%)	6.3	0.01	5.7	0.1

**Table 3:** Previous studies conducted on rabbit models investigating ceramic coating methodologies. Risedronate Calcium Phosphate Silicate Cements (RA-CPSC), Mesoporous silica (SBA15), monocalcium phosphate (MCP), polymerase chain reaction (PCR), Parathyroid hormone-related protein (PTHrP), Adiponectin (APN), Hydroxyapatite (HA), phosphate-buffered saline (PBS), TiO2 nanotube (TNT), hydroxyapatite-TiO2 nanotube (TNT-HA), calcium phosphate ceramic (CPC), poly(ε-caprolactone) (PCL), tricalcium phosphate 70% (TCP).

Author, Year	Rabbit Model	Rabbits (n) and Implant Site	Materials	Follow-Up	Type of Analyses
Gong et al., 2016 [58]	30 New Zealand rabbits	Right medial tibia epiphysis of the (*d* = 6 mm, ø = 3 mm)	Calcium silicate powder, RA-CPSC, MCP, Ca(H_2_PO_4_)2, and risedronate added into calcium silicate powder and homogeneously mixed, respectively.	8, 10 weeks	X-ray semi-quantitative analysis; PCR
Lozano et al., 2012 [59]	Osteoporosis induced rabbits	Medial and lateral distal femoral epiphysis	SBA15 and SiO_2._ The surface was chemically modified with an organic modification of silica walls with alkoxysilane, *n*-octyltriethoxysilane and functionalized by soaking the mesoporous in a solution of PTHrP in PBS.	2 weeks	Histology; Immunohistochemistry
Luo et al., 2012 [60]	60 New Zealand rabbits	Sub-periosteal mandibular (4 mm × 5 mm × 10 mm)	Porous commercial HA was physically functionalized in surface with or without APN or Matrigel or combination of both.	4 weeks	Micro-CT; Biomechanical analyses
Plaza et al., 2016 [61]	42 New Zealand white rabbits	Medial femoral condyles A	Physical incorporation of fibronectin in HA bulk material by adding HA to a fibronectin solution of in PBS.	1, 2, 5 days	Micro-CT; Histology
Shen et al. 2016 [62]	43 New Zealand white rabbits	Femoral epiphysis	TNT were immersed in supersaturated Ca(OH)_2_ solution, Ca(NO_3_)_2_·4H_2_O (0.2 M) and (NH4)2HPO4 (0.2 M) solutions to create a coating of HA. TNT-HA was subsequently functionalized with Aln by immersion in Aln solution (physical absorption).	12 weeks	X-rays; Micro-CT; Biomechanical analyses; Histology
Wu et al., 2015 [63]	16 New Zealand White rabbits	Distal femur	Strontium enriched CPC in the solid phase and PCL.	24 weeks	Micro-CT
Yu et al., 2017 [64]	40 New Zealand white rabbits	Two implants (2 mm diameter, 10 mm depth) into each femur	Ti–6Al–4V implants (ø10 × 2 mm) coated by means of plasma-spray technique with HA or CaSiO_3_ or zinc-modified calcium silicate (Ca_2_ZnSi_2_O_7_) at two different Zn contents.	4, 8, 12 weeks	Micro-CT; Histology
Gunnella et al., 2017 [65]	30 California rabbits	Not specified (5 mm wide and 4 mm)	A composite material of HA/TCP granules with or without Sr substitution.	12 weeks	Histomorphometry; Immunohistochemistry

## Data Availability

The data presented in this study are available on request from the corresponding author.

## Appendix A

**Table A1:** Ossification potential of titanium implants at 6- and 12-weeks (BIC: bone implant contact; OIC: osteoid implant contact).

Endpoint		6 Weeks	12 Weeks
Lateral	BIC (%)	3.1 ± 4.1	4.9 ± 4.6
	OIC (%)	0	−1.4 ± 0.8
Distal	BIC (%)	11.5 ± 2.3	14.1 ± 8.1
	OIC (%)	1.5 ± 3.0	5.4 ± 4.1
Medial	BIC (%)	5.3 ± 3.4	7.5 ± 2.1
	OIC (%)	−0.3 ± 0.7	3.3 ± 2.0
Proximal	BIC (%)	10.9 ± 9.1	12.1 ± 3.7
	OIC (%)	5.0 ± 0.3	4.9 ± 1.9
Overall	BIC (%)	34.6 ± 15.1	39.9 ± 19.3
	OIC (%)	6.3 ± 2.4	11.1 ± 6.5
